# Structural Characterization and Antioxidant Activity of a Crude Polysaccharide from *Cannabis sativa* Leaves

**DOI:** 10.3390/foods15101649

**Published:** 2026-05-09

**Authors:** Zhen Wang, Zhihan Shu, Qun Li, Yixin Shi, Kai Mao, Zichao Wang

**Affiliations:** 1 Shanxi Institute for Functional Food, Shanxi Agricultural University, Taiyuan 030031, China; 2 School of Biological Engineering, Henan University of Technology, Zhengzhou 450001, China

**Keywords:** *Cannabis sativa*, polysaccharide, antioxidant activity

## Abstract

As a traditional Chinese herbal medicine, *Cannabis sativa* holds broad prospects for application in the development of functional foods, pharmaceutical formulations, dietary supplements, and cosmetic products. However, the bioactivity of polysaccharides in *C. sativa* has been largely overlooked. In this study, crude *C. sativa* leaf polysaccharide (CSLP) was extracted using the hot-water extraction and ethanol-precipitation method. CSLP contains 64.15 ± 1.96% carbohydrates and 2.13 ± 0.47% protein, with a yield of 6.71 ± 0.84% (*w*/*w*). Preliminary structural characterization showed that CSLP was mainly composed of arabinose, galactose, and glucose, with a molecular weight of 28.867 kDa. CSLP not only demonstrated potential in vitro antioxidant activity against ABTS, DPPH, superoxide anion, and hydroxyl radicals, but also repaired H_2_O_2_-induced oxidative damage in RAW 264.7 macrophages by increasing the cellular levels of SOD, CAT, and GSH-Px, and reducing MDA levels. Mechanistically, CSLP possibly modulated the Nrf2/Keap1 signaling pathway in H_2_O_2_-stimulated RAW 264.7 cells via upregulating the gene expressions of *Nrf2*, *NQO1*, and *HO-1*, while downregulating *Keap1* expression. These results suggest that CSLP could potentially be used as an antioxidant ingredient in the food, pharmaceutical, and cosmetic industries.

## 1. Introduction

Hemp (*Cannabis sativa* L.), a member of the *Cannabaceae* family, is a plant with both medicinal and edible properties [1]. *C. sativa is* primarily cultivated in China, Central Asia, the Philippines, and Europe. Data from the World Food and Agriculture Organization indicate that China’s industrial *C. sativa* cultivation accounts for nearly 50% of global production [2]. *C. sativa* and its extracts exhibit various bioactivities, showing broad application prospects in functional foods, pharmaceutical formulations, dietary supplements, and cosmetics [3,4]. For instance, Roshan et al. [5] and Vozza Berardo et al. [6] reported that cannabinoids and resins isolated from *C. sativa* possess antibacterial activity against methicillin-resistant *Staphylococcus aureus* and phytopathogenic fungi. Benkirane et al. [7] and Prabsangob et al. [8] found that phenolic compounds and pectin extracted from *C. sativa* demonstrate potent antioxidant effects. Dobrucka et al. [9,10] showed that polysaccharide-based films containing *C. sativa* cannabidiol could extend food shelf-life. Charles et al. [11] and El-Sohaimy et al. [12] indicated that *C. sativa* cannabidiol and protein act as viable nutraceutical and nutritional additives. The agricultural and food applications of *C. sativa* and its seeds have also yielded favorable results [13,14]. In-depth exploration of *C. sativa* thus promises substantial benefits for human health.

Polysaccharides, macromolecules composed of more than ten monosaccharide units linked by glycosidic bonds [15,16,17,18], are ubiquitous in plants. Beyond serving as structural or energy components (e.g., starch and cellulose), plant non-starch polysaccharides frequently exhibit remarkable biological activities, including antioxidant [19,20,21,22], hypoglycemic [23,24,25], hypolipidemic [26,27], anti-aging [28,29,30], anti-inflammatory [31,32,33,34], antitumor [35,36], immunomodulatory [37,38,39] and probiotic [40,41,42,43,44], as well as antibacterial activities [45,46,47,48,49,50]. Due to their potentially high efficacy, low toxicity, and biocompatibility, these non-starch polysaccharides have attracted increasing attention across academic and industrial fields as a recognized source of natural antioxidants [51]. The structure, antioxidant activity, and therapeutic effects of plant polysaccharides have become a major research focus, as their diverse bioactivities are intrinsically linked to their antioxidant properties [52]. Specifically, polar functional groups (e.g., hydroxyl, carboxyl, and aldehyde groups) on the polysaccharide molecular surface actively participate in antioxidant defense [53]. Previous studies have explored *C. sativa* polysaccharides; for instance, hemp polysaccharides have been utilized to enhance the water solubility and expand the application of cannabidiol [54]. Current research also covers the extraction, purification, and bioactivity evaluation of hemp seed polysaccharides [55,56], along with the extraction of pectin polysaccharides from *C. sativa* fibers for use as food additives [57]. However, the antioxidant activity of *C. sativa* leaf polysaccharides remains largely uninvestigated.

To address this gap, the present study employed a hot-water extraction method to isolate crude polysaccharides from *C. sativa* leaves (CSLP), systematically characterizing their basic components and structural features. Concurrently, the bioactivity of CSLP was evaluated with a focus on its in vitro free radical-scavenging capacity and antioxidant effects in an H_2_O_2_-stimulated RAW 264.7 macrophage model. RAW 264.7 macrophages are easily induced to generate abundant reactive oxygen species (ROS) under oxidative stress, directly reflecting the protective effects of bioactive compounds against cellular oxidative damage and serving as a classic in vitro model for antioxidant evaluation [58,59]. Because ROS act as key signaling molecules for Keap1/Nrf2 pathway activation, numerous studies confirm this pathway’s involvement in polysaccharide-regulated antioxidant mechanisms [60,61,62]. We hypothesized that the antioxidant activity of CSLP is possibly mediated through this specific signaling pathway. To test this, the mRNA expression levels of *HO-1*, *NQO1*, *Keap1*, and *Nrf2* were measured in H_2_O_2_-injured RAW 264.7 cells. These findings aim to provide a theoretical basis for the advanced development and utilization of *C. sativa* and its polysaccharides.

## 2. Materials and Methods

### 2.1. Materials and Reagents

*Cannabis sativa* was harvested in Loufan County, Taiyuan City (Shanxi Province, China) and authenticated by Professor Kai Mao (Shanxi Agricultural University). After harvesting, the *C. sativa* samples were naturally stored in a cool, room-temperature environment for approximately six months before the leaves were collected for experimental use. 1,1-Diphenyl-2-picrylhydrazyl (DPPH) was purchased from Shanghai Yuelang Biotechnology Co., Ltd. (Shanghai, China). 2,2′-Azino-bis(3-ethylbenzothiazoline-6-sulfonic acid) diammonium salt (ABTS) was obtained from Jiangsu Bosite Chemical Technology Co., Ltd. (Nantong, China). Phosphate-buffered saline (PBS) and a penicillin-streptomycin mixture were purchased from Solarbio Science & Technology Co., Ltd. (Beijing, China). DMEM high-glucose medium was sourced from Shanghai Zeyi Biotechnology Co., Ltd. (Shanghai, China). The RAW 264.7 cell line was purchased from the American Type Culture Collection (ATCC) and maintained in our laboratory. The CCK-8 assay kit was purchased from Beijing Boxun Biotechnology Co., Ltd. (Beijing, China). Fetal bovine serum was obtained from Nanjing Senbeijia Biotechnology Co., Ltd. (Nanjing, China). Superoxide dismutase (SOD, S0101S), catalase (CAT, S0051), glutathione peroxidase (GSH-Px, S0057S), and malondialdehyde (MDA, S0131) assay kits (BioReagent grade) were purchased from Beyotime Biotechnology Co., Ltd. (Shanghai, China). Anhydrous ethanol, chloroform, n-butanol, ascorbic acid (defined as Vc), potassium persulfate (K_2_S_2_O_8_), Tris-HCl, resorcinol, ferrous sulfate (FeSO_4_), and other analytical grade reagents were purchased from Tianjin Kemiou Chemical Reagent Co., Ltd. (Tianjin, China).

### 2.2. Extraction of Cannabis sativa Leaves Polysaccharide

Deionized water (500 mL) was added to dried *C. sativa* leaf samples (100 g) at a liquid-to-solid ratio of 5 mL/g. The mixture was stirred at 120 rpm and 60 °C for 5 h, after which the solution was filtered through eight layers of gauze. This extraction process was repeated twice, and the resulting filtrates were pooled. The solution was then centrifuged at 8000× *g* for 10 min at room temperature to remove the insoluble residues, and the supernatant was collected. The supernatant was concentrated to approximately 200 mL using a rotary evaporator at 65 °C under 0.1 MPa vacuum. After a second centrifugation at 8000× *g* for 10 min to ensure clarity, five volumes of ethanol were added to the supernatant, and the mixture was allowed to stand overnight at 4 °C for precipitation. The resulting precipitate was collected by discarding the ethanol supernatant and redissolving it in 200 mL of deionized water. Residual ethanol was removed via distillation (65 °C, 0.1 MPa), followed by the addition of three volumes (600 mL) of Sevag solution (chloroform: n-butanol = 4:1, *v*/*v*). The mixture was shaken vigorously and centrifuged at 8000× *g* for 10 min to remove proteins. This deproteinization step was repeated 5–6 times until no white protein layer was visible. Residual organic solvents were removed by rotary evaporation (60 °C, 0.1 MPa). The polysaccharide solution was then desalted using a dialysis bag (MWCO: 10,000 Da) against deionized water for 48 h, with the water replaced every 4 h. Finally, the dialysate was lyophilized to obtain the *C. sativa* leaf polysaccharide (CSLP).

### 2.3. Basic Component Analysis

Following the methods previously reported by Wang et al. [34], the total sugar content of CSLP was determined using the phenol-sulfuric acid method with glucose as the standard (standard curve range: 0–1.0 g/L). Briefly, 0.5 mL of the sample solution was mixed with 0.5 mL of 5% (*w*/*v*) phenol solution, followed by the addition of 1.5 mL of concentrated sulfuric acid. After thorough mixing, the reaction was allowed to proceed at room temperature for 25 min, and the absorbance was measured at 490 nm. The total protein content was determined by the Coomassie Brilliant Blue method, using bovine serum albumin as the standard (standard curve range: 0–1000 μg/mL). For this, 20 μL of the sample solution was reacted with 1 mL of Coomassie Brilliant Blue solution for 5 min at room temperature, and the absorbance was recorded at 595 nm.

### 2.4. Monosaccharide Composition and Molecular Weight Analysis

For monosaccharide analysis, approximately 5 mg of CSLP was hydrolyzed in 10 mL of 2 mol/L trifluoroacetic acid (TFA) at 120 °C for 2 h. The hydrolysate was dried under nitrogen flow, washed three times with methanol, redissolved in ultrapure water, and transferred to a chromatography vial. Analysis was performed via high-performance anion-exchange chromatography with pulsed amperometric detection (HPAEC-PAD) using a Dionex CarboPac PA20 column (150 × 3.0 mm, 10 μm; Thermo Fisher Scientific, Waltham, MA, USA). The injection volume was 5 μL, the flow rate was 0.5 mL/min, and the column temperature was 30 °C. Gradient elution employed ultrapure water (A), 0.1 mol/L NaOH (B), and 0.1 mol/L NaOH containing 0.2 mol/L NaAc (C) as mobile phases. For molecular weight determination, CSLP was dissolved in 0.1 mol/L NaNO_3_ to a final concentration of 1 mg/mL, filtered through a 0.45 μm membrane, and analyzed using an Ultrahydrogel™ Linear column (300 mm × 7.8 mm). The column temperature was maintained at 45 °C, with an injection volume of 100 μL and a flow rate of 0.6 mL/min using 0.1 mol/L NaNO_3_ as the mobile phase. The system was calibrated with narrow-distribution dextran standards. Both monosaccharide and molecular weight analyses were conducted by Shanghai Sanshu Biotechnology Co., Ltd. (Shanghai, China).

### 2.5. Fourier Transform Infrared (FT-IR) Spectroscopy Analysis

Briefly, 1 mg of CSLP was mixed with 100 mg of potassium bromide (KBr) and ground thoroughly in an agate mortar before being compressed into a pellet. The functional groups were analyzed using a Nexus 470 FT-IR spectrophotometer (Nicolet, MN, USA) across a scanning range of 500–4000 cm^−1^.

### 2.6. Nuclear Magnetic Resonance (NMR) Spectroscopy Analysis

In brief, 5 mg of CSLP was dissolved in 5 mL of D_2_O and allowed to stand overnight at room temperature. The solution was then centrifuged at 8000× *g* for 5 min, and the supernatant was transferred to an NMR tube. Spectra were acquired using a Bruker AVANCE III 500 MHz spectrometer (Bruker Inc., Berlin, Germany) at 298 K. For ^1^H NMR, data were collected using a standard zg30 pulse sequence with 32 scans, a 2.0 s relaxation delay, and a 10 ppm spectral width. For ^13^C NMR, a standard zgpg30 pulse sequence was used with 8192 scans, a 3.0 s relaxation delay, and a 200 ppm spectral width.

### 2.7. In Vitro Antioxidant Activity Analysis

Based on our previous protocols [21], CSLP was dissolved in deionized water to concentrations of 0.3125, 0.625, 1.25, 2.5, and 5.0 mg/mL and filtered through a 0.22 μm aqueous membrane. The scavenging activities against DPPH, ABTS, hydroxyl, and superoxide radicals were determined according to reported methods [63] with ascorbic acid (defined as Vc) serving as the positive control. Detailed procedures are provided in the Supplementary Materials.

### 2.8. Cytotoxicity Assay

RAW 264.7 cells were cultured in DMEM supplemented with 10% (*v*/*v*) fetal bovine serum, 100 µg/mL streptomycin, and 100 U/mL penicillin at 37 °C in a 5% CO_2_ incubator. Cells were passaged using a 0.25% trypsin-EDTA solution. CSLP was prepared in DMEM at various concentrations (0.3125–5 mg/mL) and filtered. Cells were seeded in 96-well plates (5 × 10^3^ cells/well) and incubated for 24 h. After washing with PBS (pH 7.4), 100 µL of CSLP solution was added per well and incubated for another 24 h, using DMEM as a control. Cell viability was then assessed using the CCK-8 assay (4 h incubation, absorbance at 450 nm) and calculated as [(A_1_ − A_0_)/(A_2_ − A_0_)] × 100%, where A_1_, A_2_, and A_0_ represent the absorbances of the experimental, control, and blank wells, respectively.

### 2.9. Construction of H_2_O_2_-Damaged RAW 264.7 Cells Model

To establish the oxidative damage model, RAW 264.7 cells (2 × 10^5^ cells/mL) were seeded into 96-well plates. Cells were treated with H_2_O_2_ at concentrations of 0.03, 0.06, 0.09, 0.12, and 0.15 mmol/L for 6 h. Cell viability was measured using the CCK-8 kit, and the H_2_O_2_ concentration resulting in approximately 50% cell survival was selected for subsequent experiments.

### 2.10. Repairing Effects of Cannabis sativa Leaves Polysaccharide on H_2_O_2_-Damaged RAW 264.7 Cells

CSLP solutions (0.3125–5.0 mg/mL) were prepared in DMEM and filtered. Logarithmic phase RAW 264.7 cells (2 × 10^5^ cells/mL) were seeded in 24-well plates. After establishing the oxidative damage model (0.09 mmol/L H_2_O_2_ for 6 h), cells were treated with 100 μL of various CSLP concentrations for 24 h. After treatment, the cells were lysed and centrifuged (8000× *g*, 10 min) to collect the supernatant. Levels of SOD, CAT, GSH-Px, and MDA were determined using ELISA kits according to the manufacturer’s instructions. Additionally, mRNA expression levels of *HO-1*, *NQO1*, *Keap1*, and *Nrf2* were analyzed by Shanghai Sanshu Biotechnology Co., Ltd. via RT-qPCR (conditions provided in Supplementary Materials). Following the method of Ren et al. [64], primer sequences (Table 1) were used, and gene expression levels were normalized to *β*-actin.

### 2.11. Statistical Analysis

All data are presented as the mean ± standard deviation from triplicate experiments. One-way analysis of variance (ANOVA) followed by Duncan’s multiple comparison test was performed using Origin 2017 software. Statistical significance was defined at *p* < 0.05, with *p* < 0.01 indicating a significant difference and *p* < 0.001 indicating a highly significant difference.

## 3. Results and Discussion

### 3.1. Chemical Composition

As shown in Table 2, the extraction yield of CSLP from *Cannabis sativa* leaves was 6.71 ± 0.84%, while its carbohydrate and protein contents were 64.15 ± 1.96% and 2.13 ± 0.47%, respectively. These results indicate that CSLP is a crude polysaccharide that may contain other bioactive compounds, such as flavonoids, amino acids, and alkaloids [65,66]. In comparison, the extraction yield of *C. sativa* seed polysaccharide (HKP) reported by Wei et al. [55] was 3.17 ± 1.30%, which is lower than both the values obtained in the present work (6.71 ± 0.84%) and the typical polysaccharide content found in hemp seeds (10–15%). The carbohydrate and protein contents in HKP were 59.02 ± 5.02% and 3.62 ± 2.13%, respectively. Although Julakanti et al. [56] did not specify the yield for *C. sativa* seed polysaccharide (HSP), the carbohydrate (53.15 ± 1.9%) and protein (5.31 ± 0.33%) contents in HSP also differ from those observed in this study. These discrepancies in extraction yield and polysaccharide composition can be attributed to several factors. Firstly, variations in plant species or the specific plant parts used for extraction significantly influence the outcomes. For instance, Qian et al. [67] demonstrated that the yield, carbohydrate, and protein contents of polysaccharides from different forms of *Rehmannia glutinosa* varied even when identical extraction methods were applied. Secondly, specific extraction conditions also play a decisive role in determining the yield and chemical composition. Chang et al. [68] verified that parameters such as extraction temperature, time, liquid-to-solid ratio, and the number of extractions all impact the yield and composition of polysaccharides from industrial hemp residues. Furthermore, Wei et al. [69] suggested that employing different extraction solvents can result in distinct chemical components and yields for *Lycium barbarum* polysaccharides. Since the chemical composition might significantly influence the physicochemical properties and bioactivities of polysaccharides [70], these relationships will be further investigated in the subsequent works.

### 3.2. Monosaccharide Composition

Variations in the types and contents of monosaccharides might affect glycosidic bond types, charge properties, connection modes, and the spatial configuration of polysaccharides, thereby influencing their physicochemical properties and biological activities [71]. As shown in Table 2, CSLP primarily contained Fuc, Rha, Ara, Gal, Glc, Xyl, Man, GlcN, GalA, and GlcA in a molar ratio of 1.52:10.20:18.15:15.56:21.89:6.21:9.75:2.69:6.99:7.02. A polysaccharide fraction previously extracted from the bast fiber of *C. sativa* was composed of Fuc, Rha, Ara, Gal, Glc, Xyl, Man, GalA, and GlcA with a molar ratio of 1.40:23.64:26.13:23.45:5.68:4.16:3.88:9.87:1.79 [57]. While these components align with the results of the present work, GlcN was not detected in that instance. Furthermore, Chang et al. [68] verified that the monosaccharides in industrial *C. sativa* residue polysaccharide (IHRP) were Fuc, Ara, Rha, Gal, Glc, Xyl, Rib, GalA, GulA, and GlcA in a molar ratio of 1.33:19.60:10.41:20.87:27.42:4.23:3.12:6.22:0.28:2.37. Compared to the CSLP analyzed in this study, Rib and GulA were present in IHRP, whereas Man and GlcN were absent.

### 3.3. Molecular Weight

While the exact mechanism of action remains to be fully elucidated, molecular weight is recognized as a critical factor regulating the physicochemical properties and bioactivities of polysaccharides [72,73]. As summarized in Table 2, the weight-average (Mw) and number-average (Mn) molecular weights of CSLP were 28.867 kDa and 14.783 kDa, respectively, with a polydispersity index of 1.953. Notably, some low-molecular-weight fractions may have been lost during the purification process, given the 10 kDa cut-off of the dialysis membrane. Due to variations in *C. sativa* sources and extraction techniques, Julakanti et al. [56] and Tang et al. [57] reported molecular weights of 864,170 Da and 232,250 Da for polysaccharides isolated from *C. sativa*, both of which are considerably higher than the values obtained in this study. Similarly, Wei et al. [55] obtained a *C. sativa* polysaccharide with a molecular weight of 42,100 Da, slightly exceeding that of CSLP. Conversely, Li et al. [54] utilized an ultrasound-assisted method to extract two polysaccharide fractions with lower molecular weights (7171 Da and 6203 Da) than those reported here. Generally, a high molecular weight might endow polysaccharides with complex spatial conformations and distinct physical properties, whereas fractions with relatively lower molecular weights often exhibit superior biological activities, particularly antioxidant capacity. For instance, Ni et al. [74] found that a low-molecular-weight (3373 Da) dandelion polysaccharide, obtained via ultrasound-assisted enzymatic extraction, demonstrated enhanced in vitro antioxidant activity. Li et al. [75] also confirmed that a low-molecular-weight (3500 Da) pumpkin polysaccharide exhibited excellent DPPH and ABTS radical-scavenging activities. Consequently, the relatively low molecular weight of CSLP might contribute to its potent antioxidant potential, which is investigated in the following sections.

### 3.4. FT-IR

**Figure 1:**
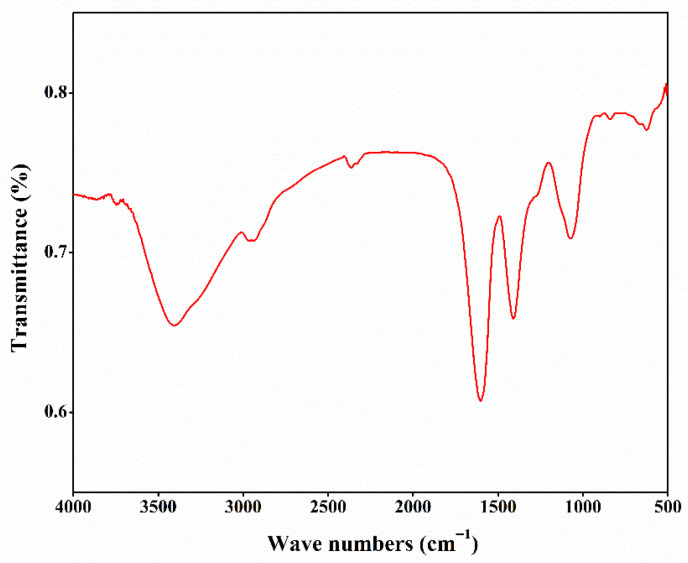
Fourier transform infrared (FT-IR) spectra of CSLP.

Fourier transform infrared (FT-IR) spectroscopy identifies the positions and intensities of absorption peaks arising from the vibrations of functional groups in a polysaccharide. As shown in Figure 1, a broad and strong absorption peak was detected at 3420 cm^−1^, indicating that CSLP might contain a wide range of -OH stretching vibrations. The absorption peak at 2937 cm^−1^ indicates the presence of C-H stretching vibrations in CSLP [63]. The signal at 1610 cm^−1^ reflects the asymmetric bending vibration of C=O, while the peak at 1400 cm^−1^ corresponds to the stretching vibration of the C-O bond [34]. Furthermore, the signal at 1067 cm^−1^ indicates the C-O-C glycosidic bond, suggesting that CSLP possesses a pyranose ring structure [39]. The absorption peak at 835 cm^−1^ suggests the possible coexistence of α- and β-glycosidic linkages, and the peak at 618 cm^−1^ is associated with the deformation vibration of C-C or C-H bonds in a cyclic structure [31]. These signals are consistent with the characteristic structural features of polysaccharides.

### 3.5. NMR

**Figure 2:**
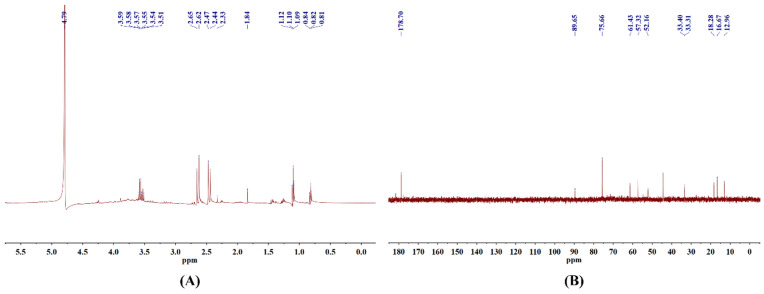
Nuclear magnetic resonance (NMR) spectra of CSLP, ^1^H NMR (**A**) and ^13^C NMR (**B**).

The chemical bonds within CSLP were further characterized using nuclear magnetic resonance (NMR) spectroscopy. In the ^1^H NMR spectrum (Figure 2A), the distinct absorption peak at δ 4.79 ppm corresponds to the chemical shift in D_2_O. The anomeric region (δ 4.3–5.5 ppm) is typically the most informative for determining α/β glycosidic linkage configurations; however, no prominent absorption peaks were detected in this region in the current study, which may limit the detailed structural characterization of the CSLP anomeric centers. Signals at δ 3.51–3.59 ppm are likely attributed to the C2–C5 protons on the pentose or hexose furan rings of the polysaccharide main chain, representing the core signals of the sugar backbone. Peaks observed at δ 2.33–2.65 ppm and δ 1.84 ppm might correspond to the methyl hydrogen signals of acetyl groups (−COCH_3_) [36]. Furthermore, signals within the range of δ 1.09–1.12 ppm indicate the presence of 6-deoxy sugar residues (such as rhamnose or fucose), originating from the methyl hydrogens at the C-6 position. Signals at δ 0.81–0.84 ppm are likely derived from methyl hydrogens at the terminus of an aliphatic side chain [38].

In the ^13^C NMR results (Figure 2B), the signal at δ 178.7 ppm is primarily attributed to the carboxyl carbon (−COOH) of GlcA. Due to the inherent limitations of the current structural analysis, only one anomeric carbon signal was clearly identified, although the anomeric region (δ 95–110 ppm) generally provides extensive information regarding complex monosaccharide compositions. The characteristic peak at δ 89.65 ppm is assigned to the anomeric carbon (C1) of the polysaccharide [21]. The signal at δ 75.66 ppm indicates the presence of β-D-Glc, while the peak at δ 61.43 ppm corresponds to the primary carbon (C-6, −CH_2_OH) of a hexose residue, confirming that CSLP contains hexose components such as Glc and Gal [37]. Additionally, signals at δ 57.32 ppm and δ 52.16 ppm may represent −CH_2_ resonances. Peaks at δ 33.31 ppm and δ 33.40 ppm suggest that CSLP might be associated with an aliphatic methylene side chain or contain a strongly electron-withdrawing group substituting a carbon on the sugar ring [38]. Finally, the three characteristic signals at δ 18.28 ppm, δ 16.67 ppm, and δ 12.96 ppm likely originate from the methyl carbons at the C-6 position of deoxy sugars.

### 3.6. In Vitro Antioxidant Activity

**Figure 3:**
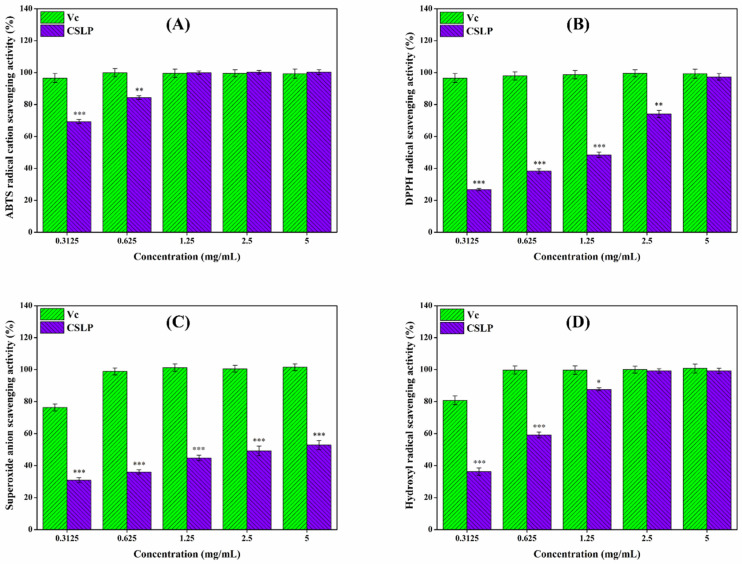
In vitro antioxidant activity of CSLP against ABTS (**A**), DPPH (**B**), superoxide (**C**) and hydroxyl (**D**) radicals. * *p* < 0.05; ** *p* < 0.01, *** *p* < 0.001 as compared to the control group of Vc.

Excessive free radicals can accelerate aging and trigger various diseases, whereas antioxidants can effectively mitigate the damage caused by these radicals to the body [76]. As illustrated in Figure 3, the in vitro antioxidant activity of CSLP exhibited a clear concentration-dependent relationship, in some instances performing comparably to the positive control, Vc. At a concentration of 5 mg/mL, the scavenging effects of CSLP against ABTS, DPPH, superoxide, and hydroxyl radicals reached 99.47 ± 1.56%, 97.31 ± 2.07%, 52.87 ± 2.90%, and 99.13 ± 1.76%, respectively (raw absorbance data for the in vitro antioxidant assays are available in the Data). Mechanistically, the hydrogen-donating ability of polysaccharides endows them with antioxidant capacities [52,53]. Furthermore, the specific types and proportions of monosaccharides might significantly influence this antioxidant activity. For instance, the presence of Ara and Gal has been shown to enhance the antioxidant activity of mung bean (*Vigna radiata* L.) skin polysaccharide [77], while high contents of Glc and GlcA are critical for the in vitro antioxidant capacity of *C. sativa* seed polysaccharide [55]. Additionally, GalA content was found to affect the antioxidant activity of polysaccharides from blue honeysuckle following probiotic fermentation [78]. Based on multiple linear regression analysis, Lo et al. [79] verified that increased Rha and Man content could enhance the antioxidant activity of *Lentinula edodes* polysaccharide. Thus, the high levels of Rha, Ara, Gal, Glc, Man, GalA, and GlcA in CSLP likely contribute to its observed antioxidant potential. Consistent with these results, Wei et al. [55] and Tang et al. [57] also confirmed that *C. sativa* polysaccharides possess free radical-scavenging abilities. Beyond composition, molecular weight plays a decisive role; lower-molecular-weight polysaccharides typically possess a relatively higher density of hydroxyl groups and a larger specific surface area, which enhances their ability to bind and react with free radicals, thereby exhibiting stronger antioxidant properties [80]. Similarly, Yu et al. [81] and Feng et al. [82] verified that a reduction in molecular weight improved the in vitro antioxidant activity of jackfruit and *Bupleurum chinense* polysaccharides, respectively.

### 3.7. Cytotoxicity Studies and Construction of H_2_O_2_-Damaged RAW 264.7 Cells Model

**Figure 4:**
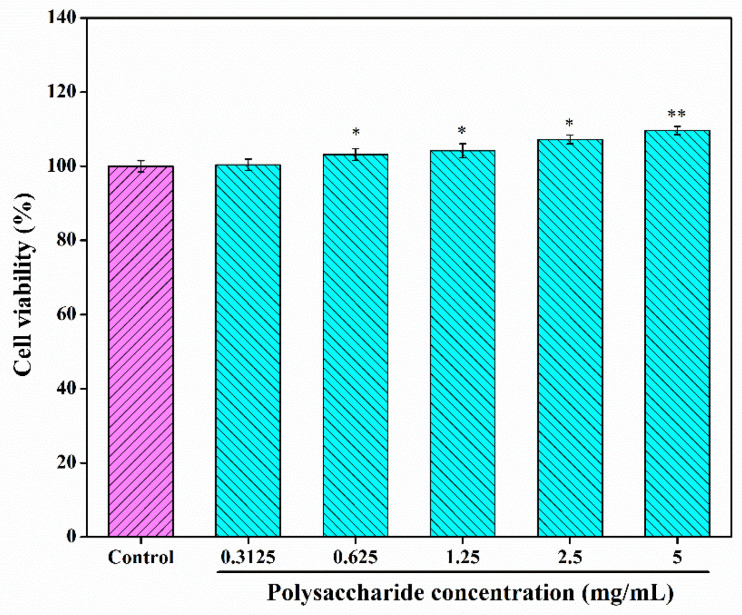
Toxicity analysis of CSLP against RAW 264.7 cells. * *p* < 0.05; ** *p* < 0.01 as compared to 100% cell activity.

To further investigate the bioactivities and potential applications of CSLP, its cytotoxicity against RAW 264.7 cells was analyzed. As shown in Figure 4, at concentrations ranging from 0.3125 to 5.0 mg/mL, the viability of RAW 264.7 cells remained above 100% and exhibited an increasing trend, reaching 109.65 ± 1.12% at the highest concentration. These results suggest that CSLP is non-toxic and safe for further investigation; moreover, it appears to promote RAW 264.7 cell proliferation within the tested concentration range.

**Figure 5:**
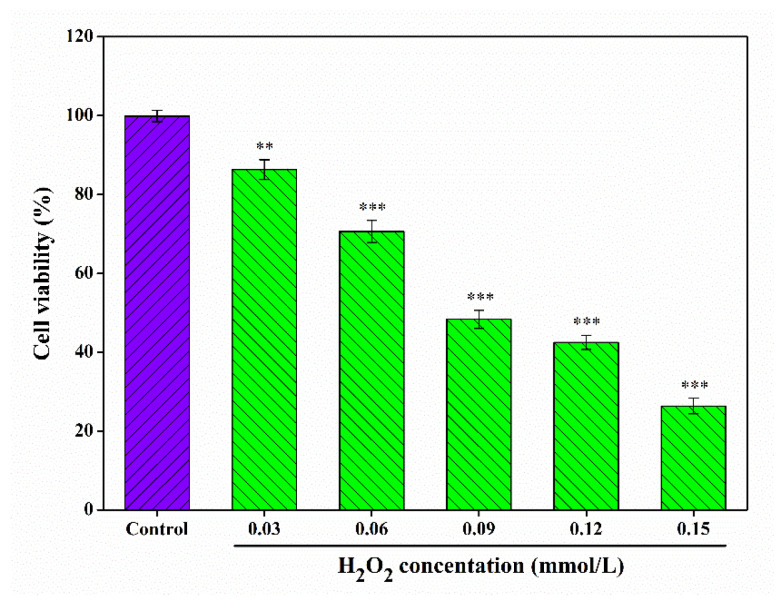
Effect of H_2_O_2_ concentration on the viability of RAW 264.7 cells. ** *p* < 0.01, *** *p* < 0.001 as compared to 100% cell activity.

In parallel, the cellular oxidative damage model was established using H_2_O_2_. As illustrated in Figure 5, the viability of RAW 264.7 cells decreased as H_2_O_2_ concentrations increased, with a viability of 48.36 ± 2.27% observed after treatment with 0.09 mmol/L H_2_O_2_ for 6 h. This survival rate confirms the successful construction of the oxidative damage model. According to Ni et al. [74], a cell survival rate of approximately 50% is considered the optimal condition for simulating oxidative damage in experimental models.

### 3.8. Repairing Effect of CSLP on H_2_O_2_-Damaged RAW 264.7 Cells

**Figure 6:**
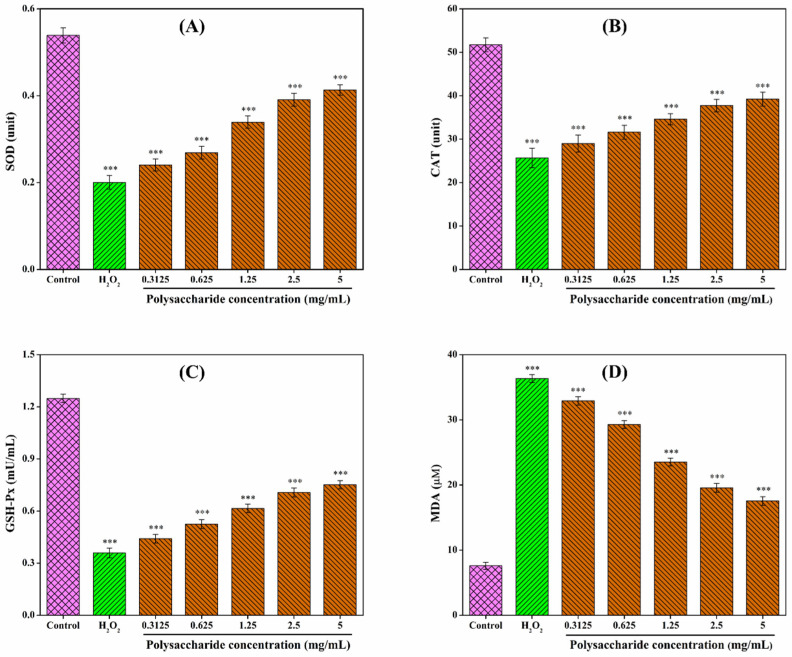
(**A**–**D**) Repairing effect of CSLP on H_2_O_2_-induced RAW 264.7 cells. *** *p* < 0.001 as compared to control group.

Oxidative stress is a critical pathogenic factor in aging and various systemic diseases [83]. Within this context, SOD, CAT, GSH-Px, and MDA serve as key indicators for assessing the degree of oxidative stress. SOD, CAT, and GSH-Px are core enzymes of the intracellular antioxidant defense system, working synergistically to eliminate reactive oxygen species (ROS). Under normal conditions, cells maintain a dynamic equilibrium between free radical-induced oxidation and antioxidant repair. However, excessive free radical accumulation disrupts this balance, leading to cellular damage [84]. Such oxidative stress triggers lipid peroxidation, resulting in the production of MDA, which subsequently stimulates phospholipase A_2_ and compromises cell membrane integrity [85]. Consequently, MDA content serves as a direct reflection of cellular oxidative damage. Following H_2_O_2_ stimulation, the levels of SOD, CAT, and GSH-Px decreased in RAW 264.7 cells, while MDA content was elevated (Figure 6). Relative to the control group (100%), the levels of SOD, CAT, and GSH-Px in the H_2_O_2_-induced model group dropped to 37.29%, 49.61%, and 28.77%, respectively, whereas the relative MDA level surged to 478.72%. CSLP treatment restored the antioxidant enzyme activities (SOD, CAT, and GSH-Px) in a concentration-dependent manner while reducing MDA levels. Upon treatment with 5.0 mg/mL CSLP, these levels were restored to 76.62%, 75.78%, 60.18%, and 231.15%, respectively, compared to the control group. These findings are consistent with results obtained by Zhou et al. [86], who demonstrated that *Pleurotus ferulae* polysaccharides enhanced antioxidant enzyme activities and reduced MDA levels in damaged RAW 264.7 cells. Similar protective effects have been reported for dandelion polysaccharides in MAC-T cells [74] and *Morchella esculenta* mycelia polysaccharides in PC12 cells [87], further validating the efficacy of CSLP in mitigating oxidative injury.

### 3.9. Effect of CSLP on Oxidative-Related Genes in RAW 264.7 Cells

**Figure 7:**
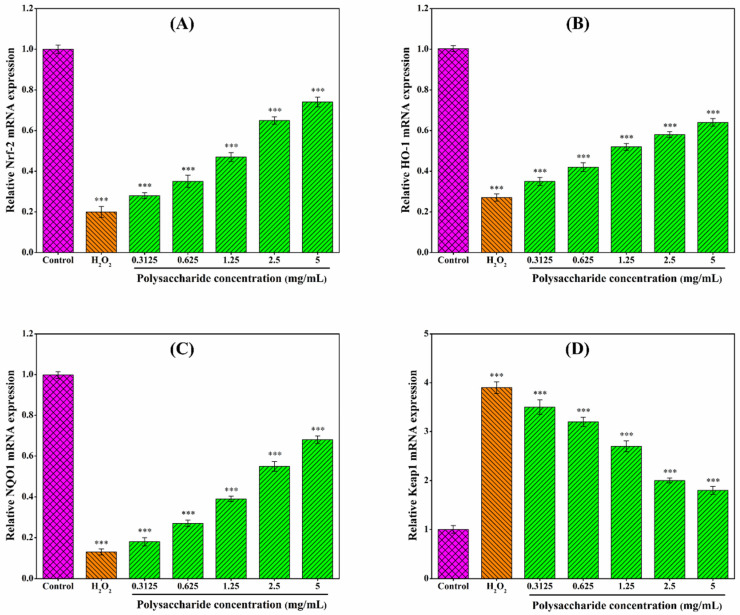
(**A**–**D**) Effects of CSLP on the mRNA expression of oxidative-related genes in H_2_O_2_-induced RAW 264.7 macrophages. *** *p* < 0.001 as compared to control group.

As shown in Figure 7, CSLP treatment upregulated the mRNA expression of *Nrf2*, *HO-1*, and *NQO1*, while downregulating *Keap1* expression in H_2_O_2_-stimulated RAW 264.7 cells in a concentration-dependent manner. Although further validation via Western blot, nuclear translocation assays, and other experimental investigations is required, these preliminary findings may suggest that the antioxidant activity of CSLP likely involves the modulation of the Nrf2/Keap1 signaling pathway. The diverse monosaccharide composition and low molecular weight of CSLP may be partially responsible for its oxidative damage-repairing effects [55,80]. Multiple studies have indicated that the protective effects of polysaccharides against oxidative damage are often mediated through the regulation of the Nrf2/Keap1 signaling pathway. For instance, Xu et al. [88] and Zhang et al. [89] suggested that polysaccharides from *Artocarpus heterophyllus* Lam. pulp and noni juice ameliorated oxidative stress in HepG2 cells by upregulating the expression of *Nrf2*, *NQO1*, and *HO-1*. Similarly, Tan et al. [90] demonstrated that *Ulva prolifera* polysaccharide alleviated H_2_O_2_-induced oxidative damage in BRL-3A cells through the upregulation of the same key genes. Furthermore, the activation of the Nrf2/Keap1 pathway as a mechanism for polysaccharide-induced oxidative stress amelioration has been confirmed in both mouse [61] and *Macrobrachium rosenbergii* [60] models. Building upon these in vitro findings, future research will focus on the digestion, in vivo biological activities, and underlying molecular mechanisms of CSLP to further explore its potential applications in the functional food and pharmaceutical industries.

## 4. Conclusions

In this study, a crude polysaccharide (CSLP) was isolated from *Cannabis sativa* leaves via hot-water extraction and ethanol precipitation, achieving a yield of 6.71 ± 0.84%. Preliminary structural characterization revealed that CSLP is a heteropolysaccharide composed of Fuc, Rha, Ara, Gal, Glc, Xyl, Man, GlcN, GalA, and GlcA in a molar ratio of 1.52: 10.20: 18.15: 15.56: 21.89: 6.21: 9.75: 2.69: 6.99: 7.02. The weight-average molecular weight (Mw) was determined to be 28.867 kDa with a polydispersity index of 1.953. Bioactivity assays demonstrated that CSLP possesses potent antioxidant capacity and mitigates H_2_O_2_-induced oxidative damage in RAW 264.7 macrophages. This protective effect is likely mediated through the modulation of intracellular antioxidant enzymes and the activation of the Nrf2/Keap1 signaling pathway. Future research should prioritize elucidating the detailed structure–activity relationships of CSLP. Furthermore, rigorous in vivo validation is essential prior to its practical application; such studies should encompass comprehensive safety assessments, clinical scoring, advanced imaging, pharmacokinetics, and histopathological evaluations. These findings provide a theoretical foundation for the development of *C. sativa* leaf polysaccharides as natural antioxidants in the functional food and pharmaceutical sectors.

## Figures and Tables

**Table 1:** The related genes and primer sequences used in present work.

Related Genes	Primer Sequences
*β-actin*	RTf-*β*-actin: 5′-TGTCCACCTTCCAGCAGATGT-3′
	RTr-*β*-actin: 5′-AGCTCAGTAACAGTCCGCCTAGA-3′
*Nrf2*	RTf-Nrf2: 5′-AGCGGATTGCTCGTAGACAG-3′
	RTr-Nrf2: 5′-TCAATCAAATCCATGTCCTTGGC-3′
*Keap1*	RTf-Keap1: 5′-ATGGCGGGGCCTCTGA-3′
	RTr-Keap1: 5′-CTCAGGGGCAGAAATTGGGT-3′
*HO-1*	RTf-HO-1: 5′-CTGAGAATGCCGAGTTCAT-3′
	RTr-HO-1: 5′-GGAAGTAGAGGGGCGTGTAG-3′
*NQO1*	RTf-NQO1: 5′-TGGTGGAGTCGGACCTCTATG-3′
	RTr-NQO1: 5′-CATGGCAGCGTAAGTGTAAGC-3′

**Table 2:** Chemical composition and structural characteristics of a *C. sativa* leaf polysaccharide (CSLP).

Parameters	CSLP
Extraction yield (dry weight, %)	6.71 ± 0.84
Carbohydrate content (*w*/*w*, %)	64.15 ± 1.96
Protein content (*w*/*w*, %)	2.13 ± 0.47
**Monosaccharide composition (μg/mg)**	
Fucose (Fuc)	1.8647
Rhamnose (Rha)	12.4853
Arabinose (Ara)	20.3126
Galactose (Gal)	20.9019
Glucose (Glc)	29.4069
Xylose (Xyl)	6.9531
Mannose (Man)	13.0963
Glucosamine (GlcN)	3.5974
Galacturonic acid (GalA)	10.1198
Glucuronic acid (GlcA)	10.1623
**Molecular weight (kDa)**	
Weight-average molecular weight (M_w_)	28.867
Number-average molecular weight (M_n_)	14.783
Polydispersity (M_w_/M_n_)	1.953

## Data Availability

The raw data supporting the conclusions of this article will be made available by the authors on request.

## Supplementary Materials

The following supporting information can be downloaded at: https://www.mdpi.com/article/10.3390/foods15101649/s1.
