# Supplementary material for: Structural Characterization and Antioxidant Activity of a Crude Polysaccharide from Cannabis sativa Leaves
Source: Foods. 2026 May 9;15(10):1649. doi: 10.3390/foods15101649 (PMC13205411; doi:10.3390/foods15101649)
Supplement: Supplementary file 1 [file foods-15-01649-s001.zip › foods-4238263-supplementary.pdf]

1 *In vitro* antioxidant activity analysis of CSLP

1.1 DPPH free radical scavenging activity

CSLP was dissolved in deionized water to prepare solutions of different concentrations (0.3125, 0.625, 1.25, 2.5, and 5.0 mg/mL) and filtered through a 0.45 μm membrane. Then, 0.1 mL of the CSLP solution was mixed with 0.1 mL of DPPH solution (2×10^{-4} mol/L). After incubation in the dark for 30 min at room temperature, the absorbance was measured at 517 nm. Ascorbic acid (defined as Vc) was served as the positive control. The DPPH radical scavenging activity was calculated using the formula: Scavenging rate (%) = $[1 - (A_1 - A_2)/A_0] \times 100\%$, where A_1 is the absorbance of the CSLP and DPPH mixture, A_2 is the absorbance of CSLP in 60% ethanol, and A_0 is the absorbance of the DPPH solution with deionized water.

1.2 ABTS free radical scavenging activity

The CSLP working solution was prepared by mixing equal volumes of 7.4 mmol/L ABTS and 2.6 mmol/L $K_2S_2O_8$ solutions, incubating the mixture in the dark at room temperature for 12 h, and then diluting it with PBS (pH 7.4) to an absorbance of 0.7 ± 0.02 at 734 nm. Then, 0.2 mL of the ABTS working solution was mixed with 0.05 mL of CSLP solution. After 6 min of incubation in the dark, the absorbance was measured at 734 nm, with Vc as the positive control. The scavenging activity was calculated as: Scavenging rate (%) = $[1 - (A_1 - A_2)/A_0] \times 100\%$, where A_1 is the absorbance of the CSLP and ABTS mixture, A_2 is the absorbance of CSLP with deionized water, and A_0 is the absorbance of the ABTS working solution with deionized water.

1.3 Superoxide anion free radical scavenging activity

Briefly, 0.25 mL of 50 mmol/L Tris-HCl (pH 8.2) was mixed with 0.04 mL of CSLP solution. The mixture was incubated at 25 °C for 10 min, after which, 0.2 mL of 7.5 mmol/L pyrocatechol solution was added. The reaction proceeded for another 20 min at 25 °C. Finally, the absorbance was measured at 320 nm, with Vc as the positive control. The scavenging activity was calculated as: Scavenging rate (%) = $[1 - (A_1 - A_2)/A_0] \times 100\%$, where A_1 is the absorbance of the complete reaction mixture, A_2 is the absorbance of CSLP in Tris-HCl, and A_0 is the absorbance of the control reaction with deionized water.

1.4 Hydroxyl radical scavenging activity

In brief, 1 mL of 6 mmol/L $FeSO_4$ solution was mixed with 1 mL of CSLP solution, followed by the addition of 1 mL of 6 mmol/L H_2O_2. The mixture was incubated at 37 °C for 10 min. Subsequently, 1 mL of 6 mmol/L salicylic acid in anhydrous ethanol was added, and the mixture was incubated for another 30 min

at 37 °C. After centrifugation (3,000 × g, 2 min), the absorbance of the supernatant was measured at 510 nm, with Vc as the positive control. The scavenging activity was calculated as: Scavenging rate (%) = [1 - (A_1 - A_2)/A_0] × 100%, where A_1 is the absorbance of the complete reaction, A_2 is the sample blank (with deionized water instead of H_2O_2), and A_0 is the reagent blank (with deionized water instead of sample).

2. RT-qPCR conditions

RAW 264.7 cells were collected and washed three times with cold PBS and the intracellular total RNA was extracted using total RNA extraction kit. In order to avoid the degradation of extracted RNA, extracted RNA was immediately reverse-transcribed into cDNA using a reverse transcription kit. The reverse transcription procedure of cDNA using following steps: 25 °C for 10 min, 50 °C for 20 min, 85 °C for 5 min, and 4 °C for 10 min. The cDNA was diluted 5 folds and then used as template for RT-qPCR amplification, and the amplification procedure was as follows: 50 °C for 2 min, 95 °C for 10 min, 95 °C for 30 s, and 60 °C for 30 s, a total of 40 cycles.
